# Supplementary material for: C9ORF72 GGGGCC repeat-associated non-AUG translation is upregulated by stress through eIF2α phosphorylation
Source: Nat Commun. 2018 Jan 4;9:51. doi: 10.1038/s41467-017-02495-z (PMC5754368; doi:10.1038/s41467-017-02495-z)
Supplement: Supplementary file 1 — Supplementary Information [file 41467_2017_2495_MOESM1_ESM.pdf]

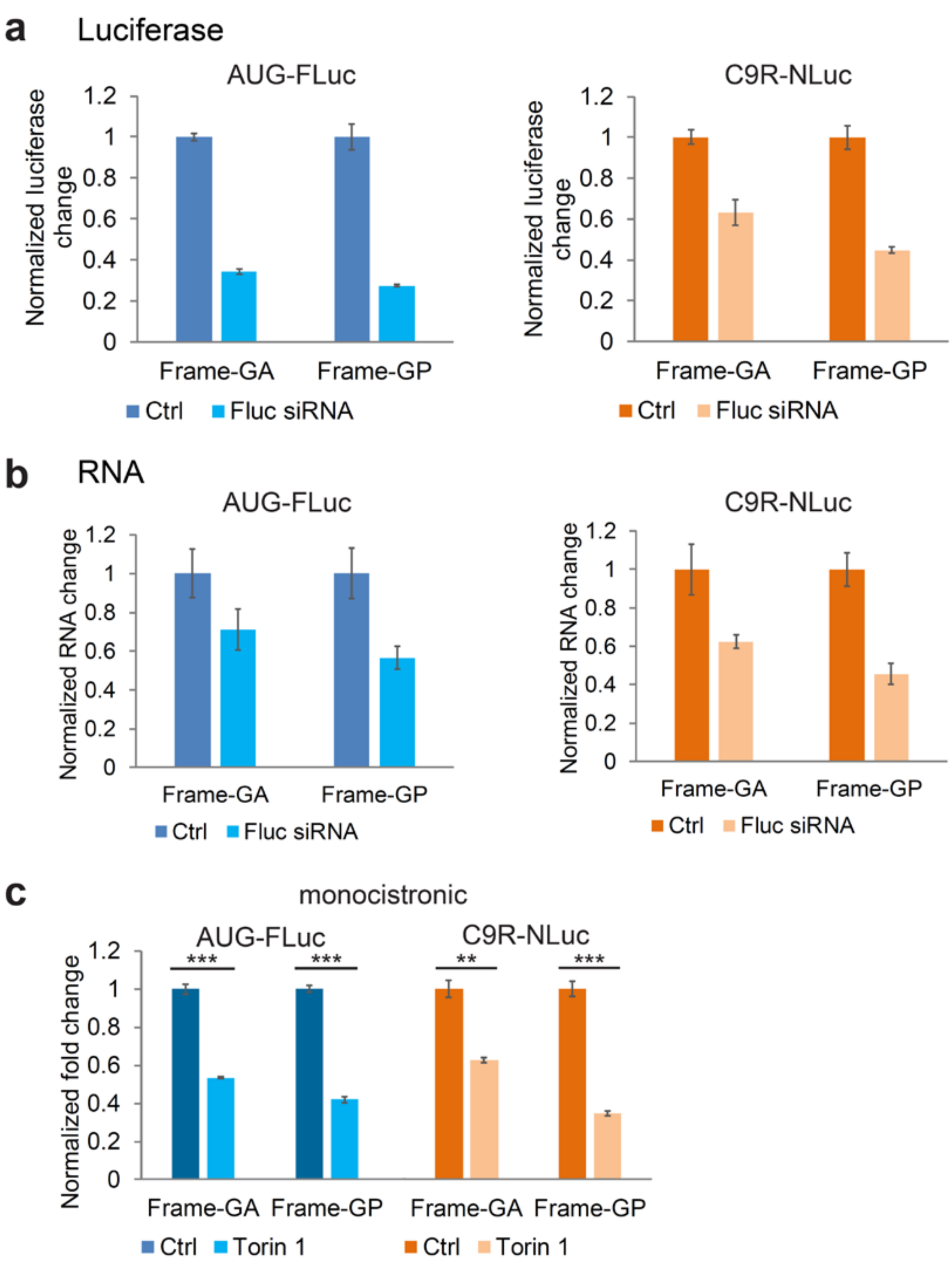

Supplementary Figure 1: Dual-luciferase reporters for RAN translation of C9ORF72 GGGGCC repeats. (a) Relative luciferase levels of AUG-FLuc (left) and C9R-NLuc (right) in the bicistronic reporter cell lines in presence of non-targeting siRNA or siRNA against Fluc. Data are mean ± s.e.m. from three biological replicates. **(b)** Relative RNA levels of FLuc (left) and NLuc (right) in the bicistronic reporter cells as above, revealed by qRT-PCR. Data are mean ± s.e.m. from three biological replicates. **(c)** Expression of AUG-FLuc and C9R-NLuc in monocistronic reporter cells under mTOR pathway inhibition by Torin 1 treatment. Data are mean ± s.e.m. from three biological replicates. **$P<0.005$, ***$P<0.0005$, two-tailed *t* test.

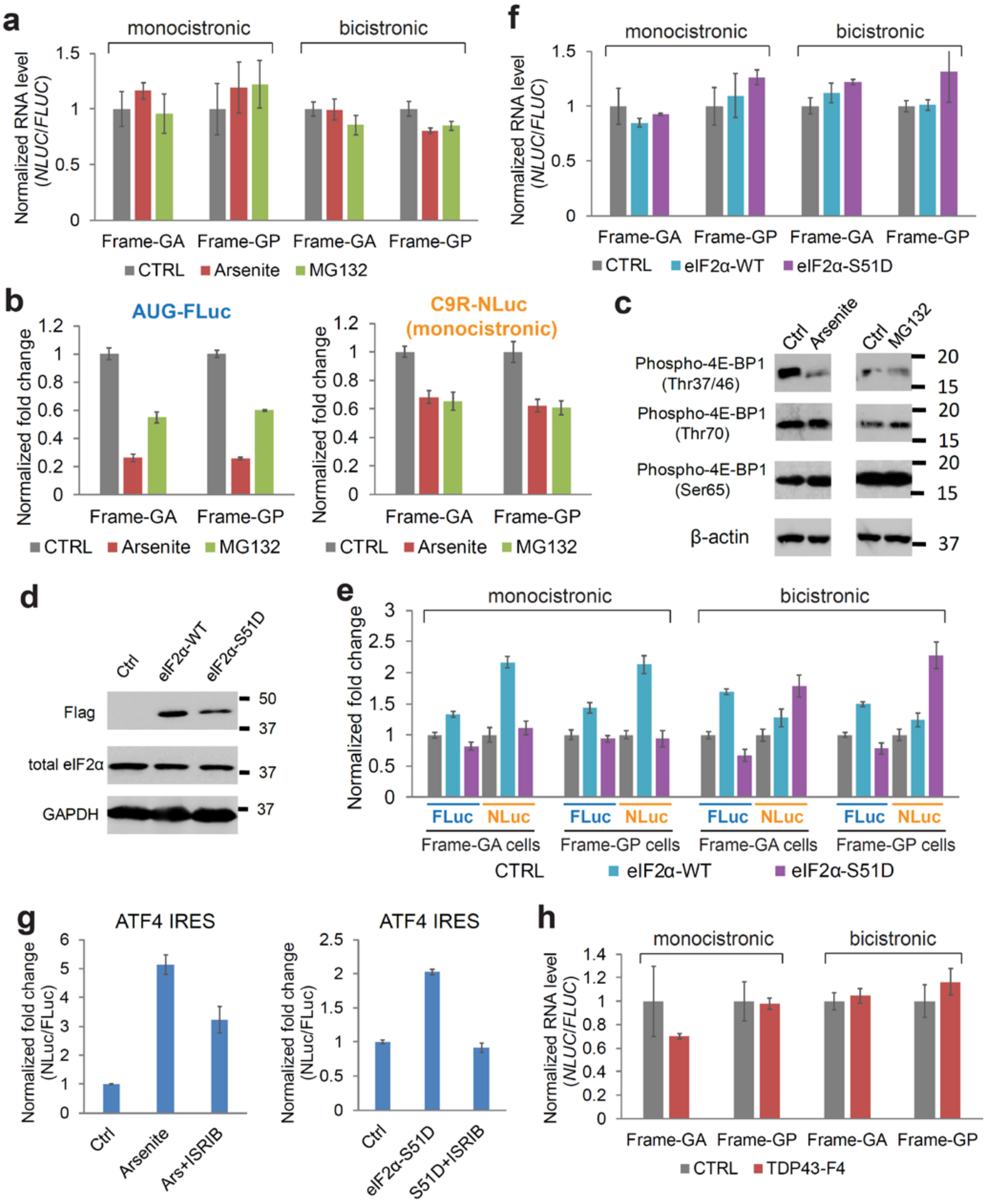
a
monocistronic
bicistronic
Normalized RNA level (NLUC/FLUC)
1.5
1
0.5
0
Frame-GA Frame-GP Frame-GA Frame-GP
CTRL Arsenite MG132
b
AUG-FLuc
Normalized fold change
1.2
1
0.8
0.6
0.4
0.2
0
Frame-GA Frame-GP
CTRL Arsenite MG132
C9R-NLuc (monocistronic)
Normalized fold change
Frame-GA Frame-GP
CTRL Arsenite MG132
c
Ctrl Arsenite Ctrl MG132
Phospho-4E-BP1 (Thr37/46)
Phospho-4E-BP1 (Thr70)
Phospho-4E-BP1 (Ser65)
β-actin
20
15
37
d
Ctrl eIF2α-WT eIF2α-S51D
Flag
total eIF2α
GAPDH
50
37
e
monocistronic
bicistronic
Normalized fold change
3
2.5
2
1.5
1
0.5
0
FLuc NLuc FLuc NLuc FLuc NLuc FLuc NLuc
Frame-GA cells Frame-GP cells Frame-GA cells Frame-GP cells
CTRL eIF2α-WT eIF2α-S51D
f
monocistronic
bicistronic
Normalized RNA level (NLUC/FLUC)
1.5
1
0.5
0
Frame-GA Frame-GP Frame-GA Frame-GP
CTRL eIF2α-WT eIF2α-S51D
g
ATF4 IRES
Normalized fold change (NLuc/FLuc)
6
5
4
3
2
1
0
Ctrl Arsenite Ars+ISRIB
ATF4 IRES
Normalized fold change (NLuc/FLuc)
2.5
2
1.5
1
0.5
0
Ctrl eIF2α-S51D S51D+ISRIB
h
monocistronic
bicistronic
Normalized RNA level (NLUC/FLUC)
1.4
1.2
1
0.8
0.6
0.4
0.2
0
Frame-GA Frame-GP Frame-GA Frame-GP
CTRL TDP43-F4

Supplementary Figure 2: eIF2α phosphorylation enhances cap-independent RAN translation under stress. (a) NLuc and FLuc RNA levels were measured by qRT-PCR without and with arsenite or MG132 stress stimuli in each reporter cell line. NLuc was normalized to FLuc levels. Error bars represent s.e.m in four biological and technical replicates. **(b)** Expression of monocistronic luciferase reporters under treatment with arsenite or MG132 stimuli. Relative expression of AUG-FLuc and C9R-NLuc reporters were compared with no stress control. The luciferase signals were normalized to the total protein amount. Data are mean ± s.e.m. from three biological replicates. **(c)** Reporter cells were treated with arsenite or MG132. Immunoblotting of phospho-4E-BP1 using antibodies recognizing different phosphorylation sites. β-actin was blotted as internal control. **(d)** Reporter cells were transfected with plasmids expressing GFP (negative control), FLAG-tagged wild type or S51D mutant of eIF2α. The relative expression levels were examined by immunoblotting using antibodies recognizing the FLAG tag and total eIF2α. GAPDH was blotted as internal control. **(e)** After 1 day of transfection, cells were induced to express translation reporters by doxycycline and luciferase activities were measured after another 24 hours. Relative expression of AUG-FLuc and C9R-NLuc reporters were compared with GFP transfection control. The luciferase signals were normalized to the total protein amount. Data are mean ± s.e.m. from three biological replicates. **(f)** NLuc and FLuc RNA levels were measured by qRT-PCR after transfection of GFP, wild type or S51D mutant of eIF2α in each reporter cell line. **(g)** Fold change of ATF4 5'UTR IRES-mediated translation with arsenite stimuli (left) or eIF2α S51D expression (right), without or with pre-treatment of ISRIB. Error bars represent s.e.m in three biological replicates. **(h)** NLuc and FLuc RNA levels were measured by qRT-PCR after transfection of GFP (negative control), or TDP43-F4 in each reporter cell line. Error bars represent s.e.m in three biological replicates.

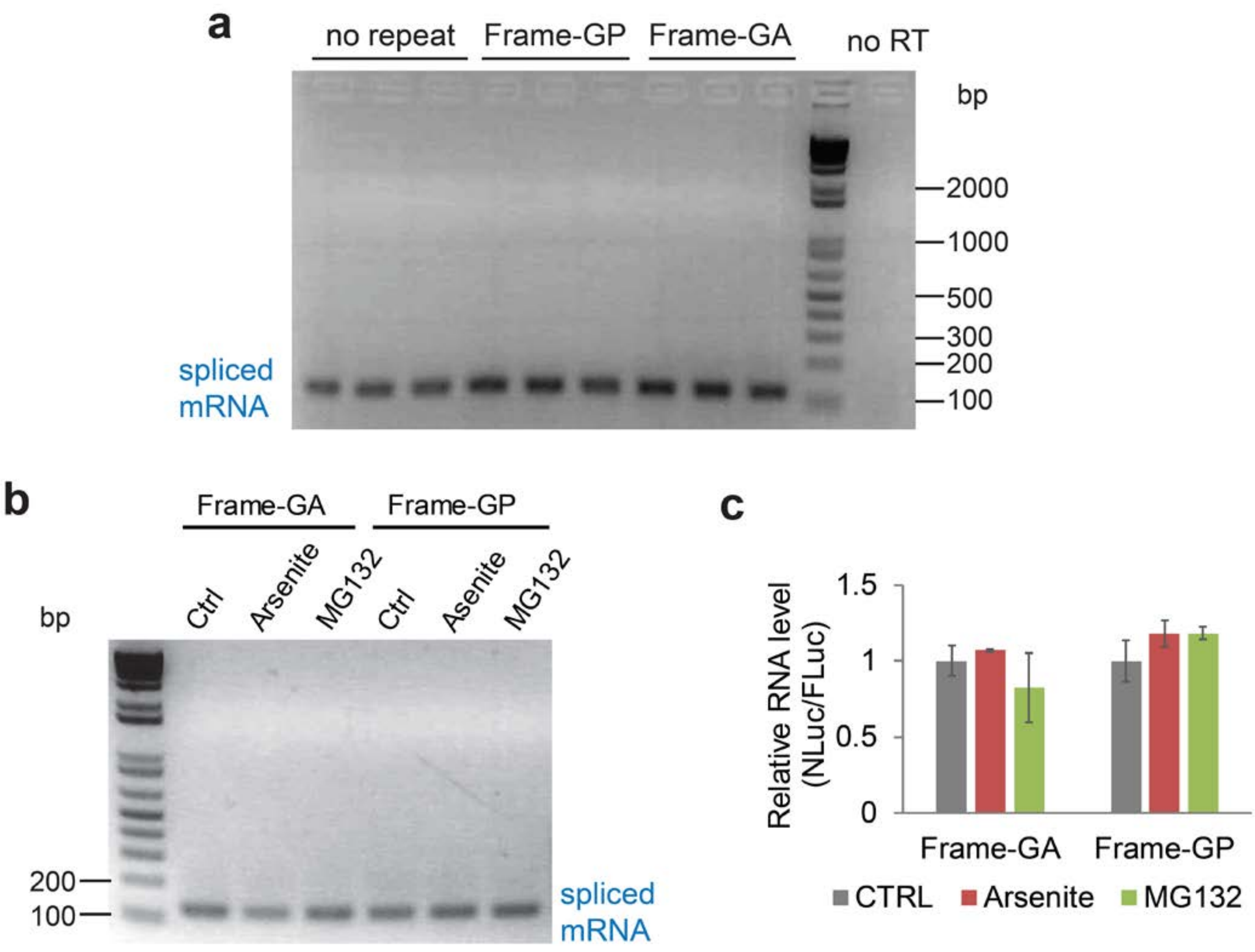

Supplementary Figure 3: RNA splicing and expression of the RAN translation reporter for intronic GGGGCC repeats. (a) PCR amplification of spliced mRNA from the reporters with and without the expanded repeats in the intron. **(b)** PCR amplification of spliced reporter mRNA upon stress stimuli. **(c)** The relative level of NLuc normalized to FLuc is not changed by stress stimuli, measured by qRT-PCR in each reporter cell line. Error bars represent s.e.m in three biological replicates.

Figure 1c
Monocistronic reporter

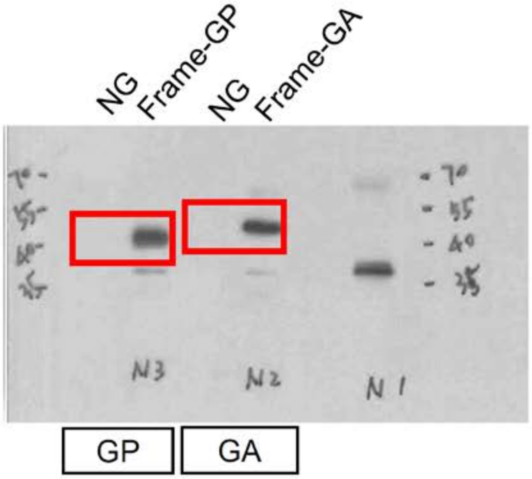

Figure 1f
Biscistronic reporter

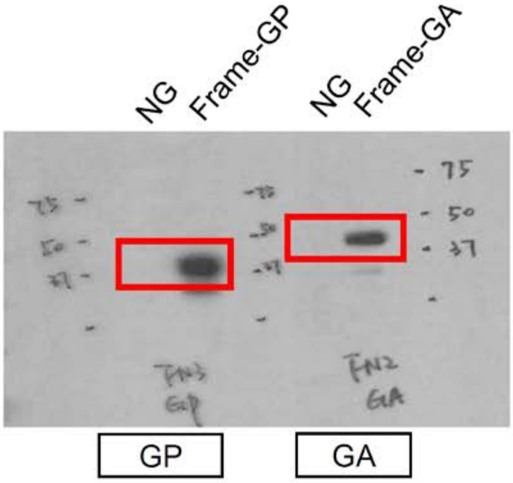

Figure 1h

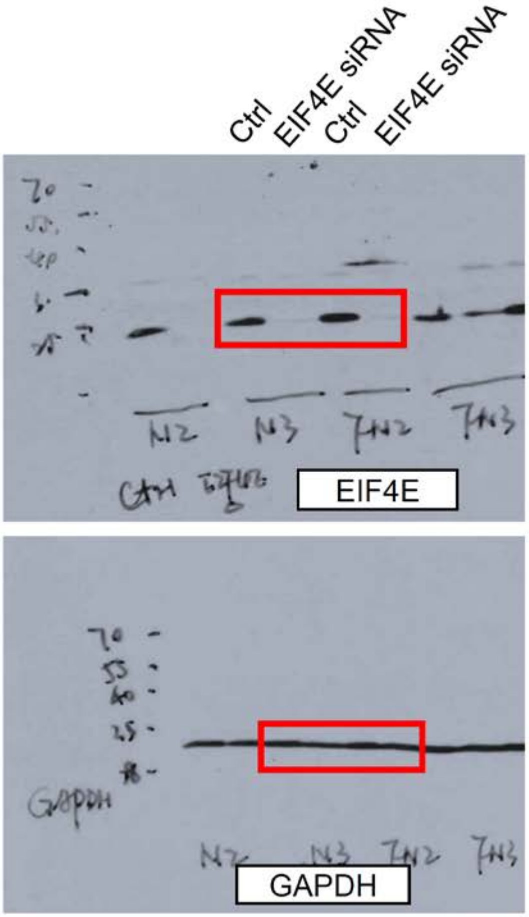

Supplementary Figure 4 (cont'd): Uncropped Western Blots shown in main figures.

Figure 1j

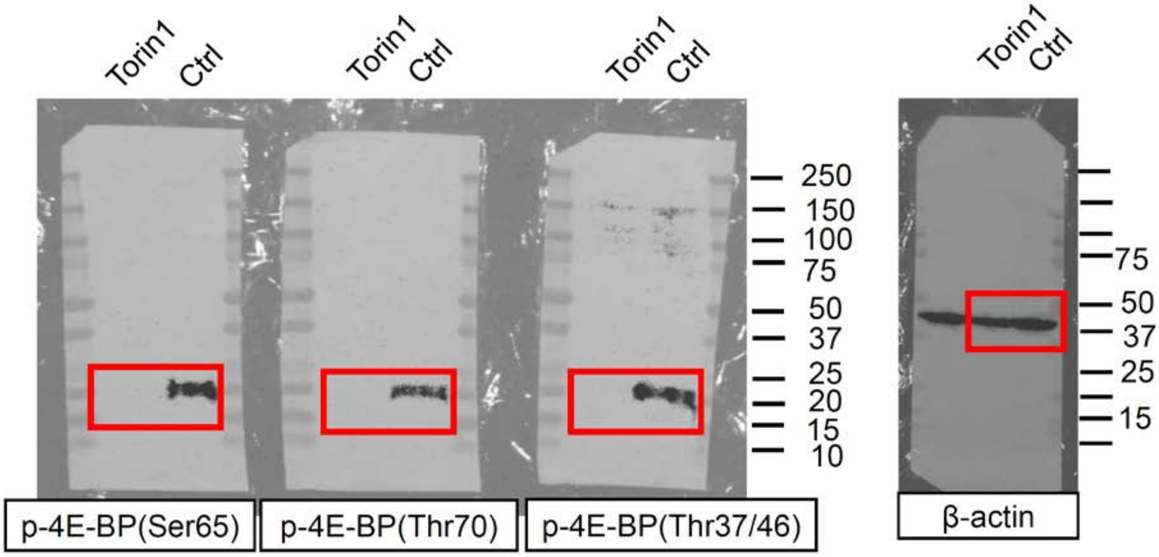

Figure 2d

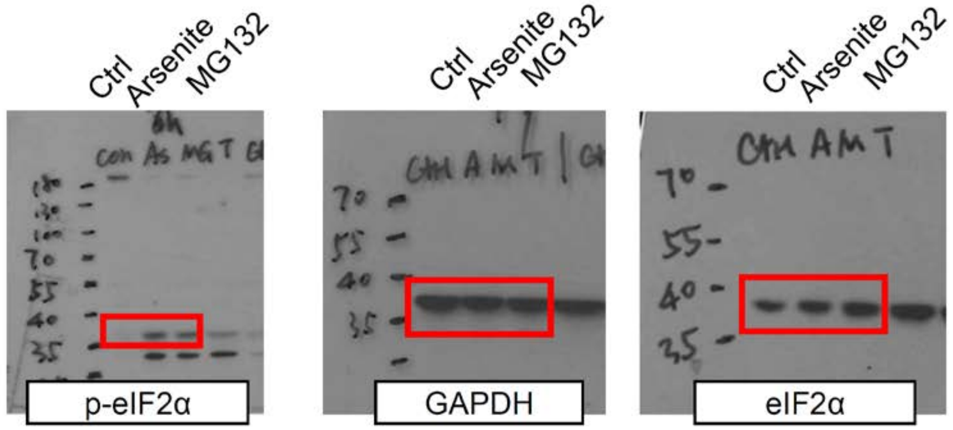

Supplementary Figure 4 (cont'd): Uncropped Western Blots shown in main figures.

Figure 4c

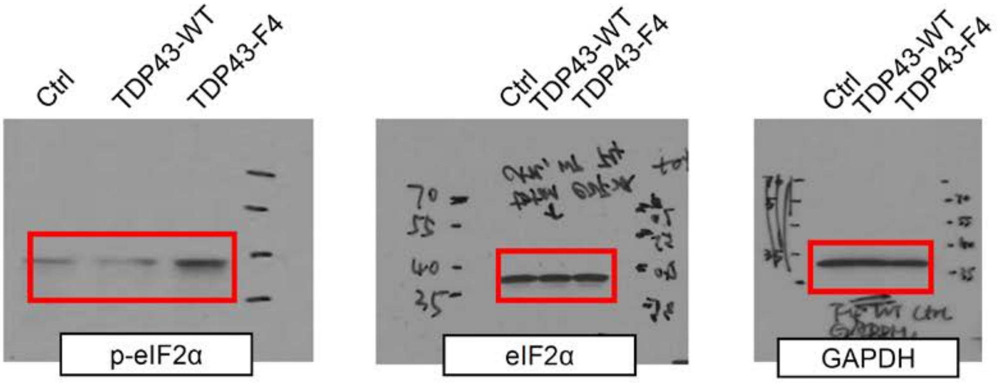

Figure 4e

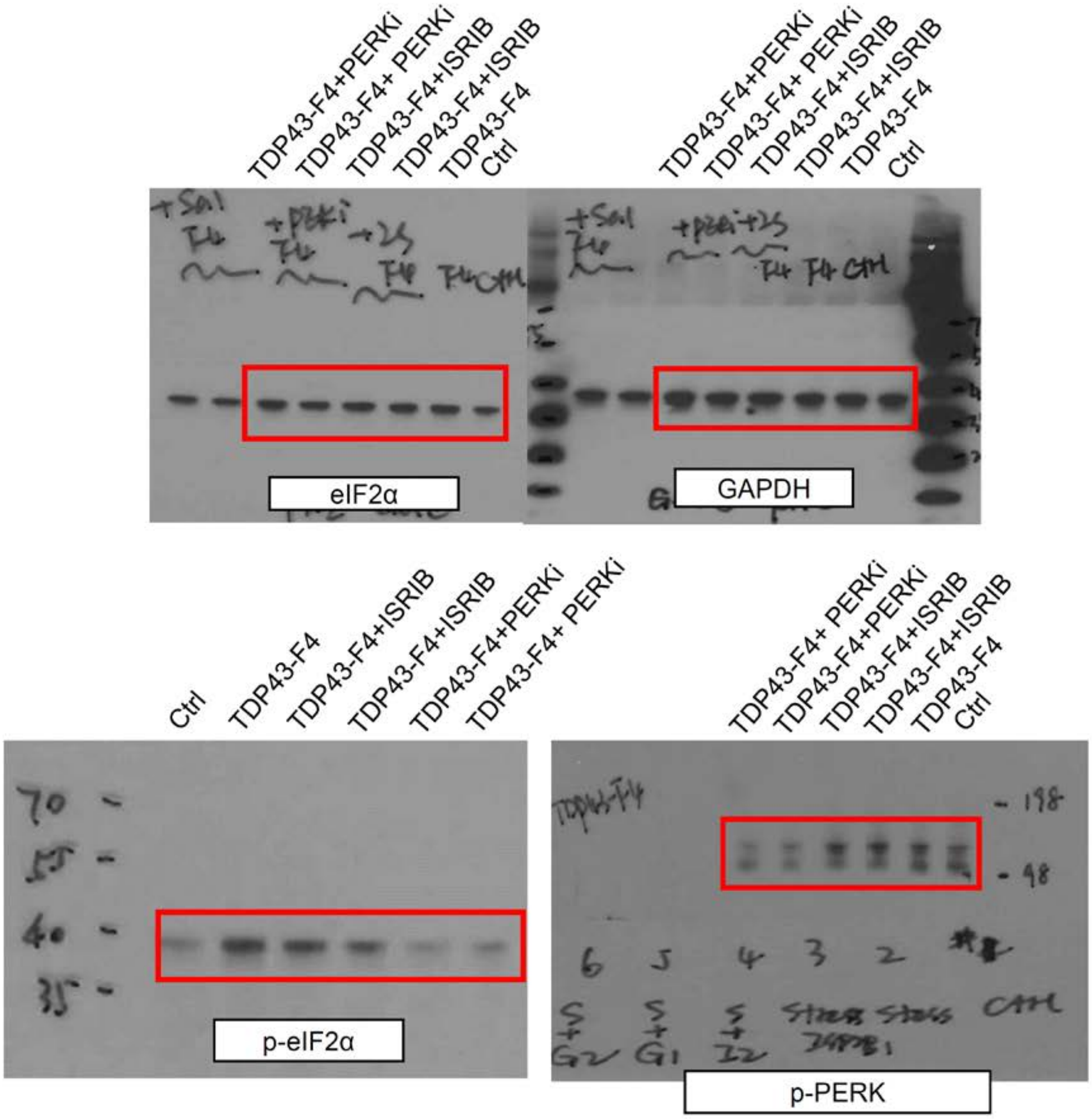

Supplementary Figure 4 (cont'd): Uncropped Western Blots shown in main figures.

Figure 5c

Bicistronic splicing reporter

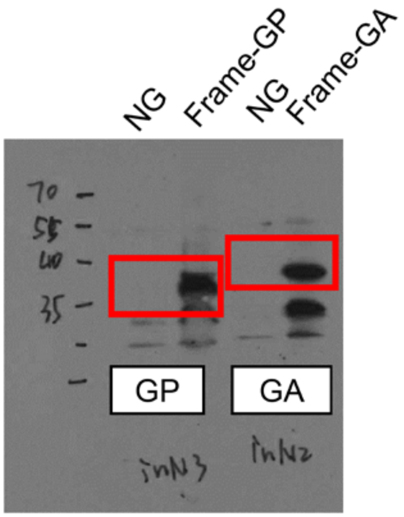

Figure 6a

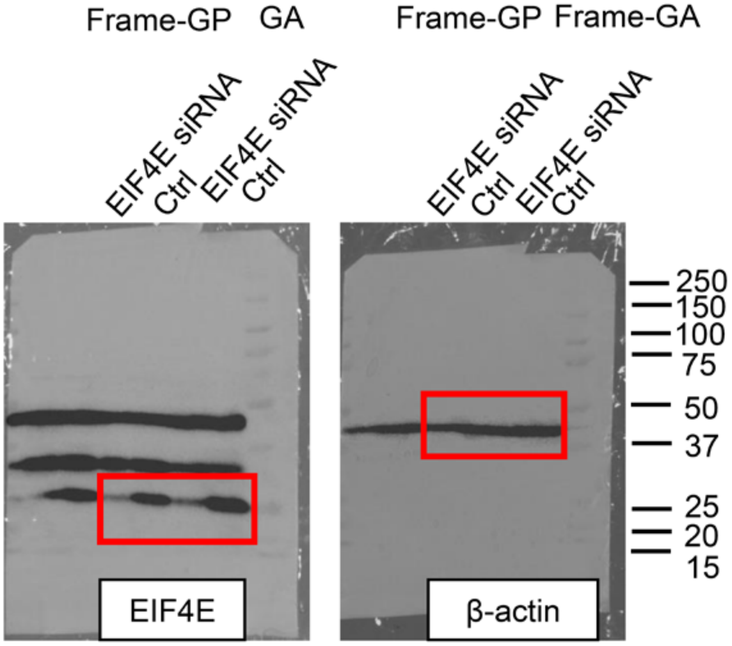

Figure 6c

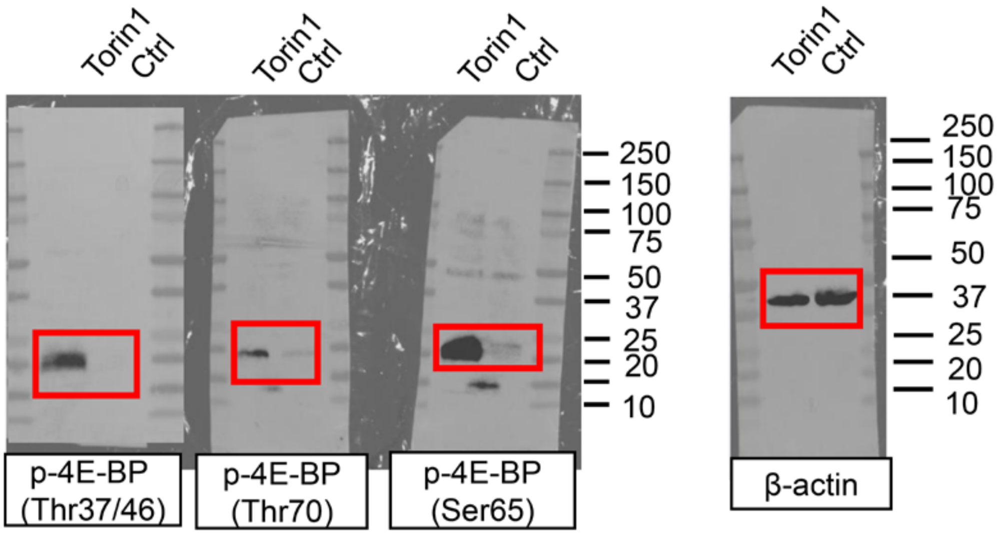

Supplementary Figure 4 (cont'd): Uncropped Western Blots shown in main figures.

Figure S2c

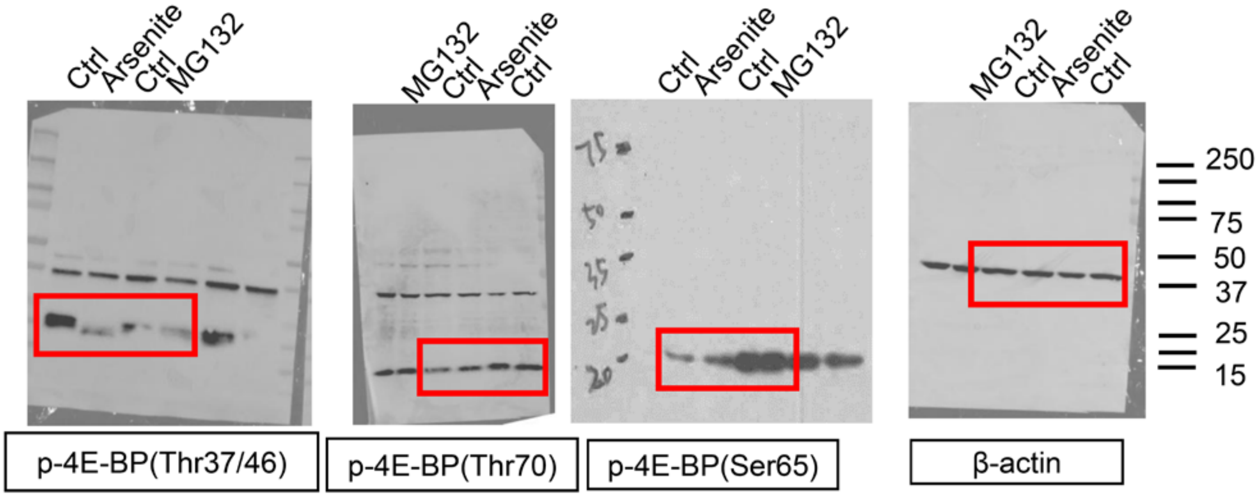

Figure S2d

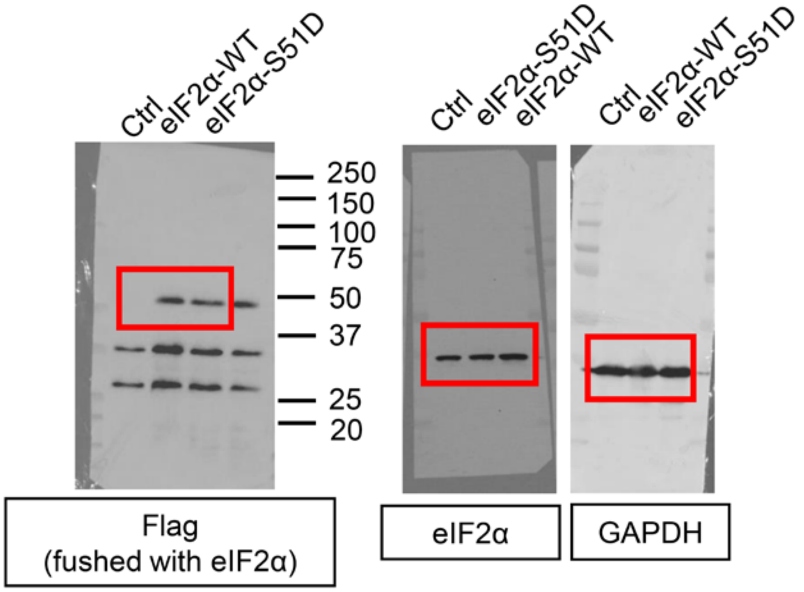

Supplementary Figure 4: Uncropped Western Blots shown in main figures.

Supplementary Table 1: qPCR primer sequences.

NLuc	Forward	5'-GTCCGTAACTCCGATCCAAAG-3'
	Reverse	5'-TGCCATAGTGCAGGATCACCT-3'
FLuc	Forward	5'-GTGACTTCCCATTTGCCACC-3'
	Reverse	5'-TGATCTGGTTGCCGAAGATG-3'
GAPDH mRNA	Forward	5'-GAGTCAACGGATTTGGTCGT-3'
	Reverse	5'-TTGATTTTGGAGGGATCTCG-3'
MTRNR1	Forward	5'-CCCTGAAGCGCGTACACACC-3'
	Reverse	5'-GTCCAAGTGCACTTTCCAGT-3'
GAPDH pre-mRNA	Forward	5'-AAGGTGAAGGTCGGAGTCAAC-3'
	Reverse	5'-GCTGACCTTGAGCTCTCCTTG-3'
reporter spliced exon	Forward	5'-GAGGTGCGTCAAACAGCGAC-3'
	Reverse	5'-TTTGGCATCTTCCCTCGAGG-3'
reporter pre-mRNA	Forward	5'-GAGGTGCGTCAAACAGCGAC-3'
	Reverse	5'-AGAGCAAGTAGTGGGGAGAG-3'
